# Universal Effectiveness of Inducing Magnetic Moments in Graphene by Amino-Type sp^3^-Defects

**DOI:** 10.3390/ma11040616

**Published:** 2018-04-17

**Authors:** Tao Tang, Liting Wu, Shengqing Gao, Fang He, Ming Li, Jianfeng Wen, Xinyu Li, Fuchi Liu

**Affiliations:** 1 College of Science & Key Laboratory of Nonferrous Materials and New Processing Technology, Guilin University of Technology, Guilin 541004, China; tingabce@163.com (L.W.); gaoshengqing@hust.edu.cn (S.G.); hefang7132@163.com (F.H.); liming928@163.com (M.L.); wjfculater@163.com (J.W.); lixinyu5260@163.com (X.L.); 2 College of Physics and Technology, Guangxi Normal University, Guilin 541004, China

**Keywords:** graphene, sp^3^-defect, amino group, magnetic moment

## Abstract

Inducing magnetic moments in graphene is very important for its potential application in spintronics. Introducing sp^3^-defects on the graphene basal plane is deemed as the most promising approach to produce magnetic graphene. However, its universal validity has not been very well verified experimentally. By functionalization of approximately pure amino groups on graphene basal plane, a spin-generalization efficiency of ~1 μ_B_/100 NH_2_ was obtained for the first time, thus providing substantial evidence for the validity of inducing magnetic moments by sp^3^-defects. As well, amino groups provide another potential sp^3^-type candidate to prepare magnetic graphene.

## 1. Introduction

The introduction of magnetic moments in graphene is a long-standing hot topic [1]. Generally speaking, the net spins in graphene come from unpaired electrons; however, all the electrons in the intrinsic graphene are compensated for owning to the π-symmetry system. Thus, breaking the symmetric structure of graphene is a feasible approach to make graphene magnetic. These approaches to introduce magnetic moments in graphene can be divided two ways [2]: (i) creating sp^3^-defects on the basal plane of graphene sheets via atoms or functional groups chemisorbed on carbon networks to form covalent sp^3^-type bonds, typically H [3], F [4] or hydroxyl group [2,5,6]; and (ii) producing edge-type defects at the edge sites via bombarding graphene sheets to introduce vacancies [4,7], cutting graphene into quantum dots [8], nanoribbons [9] or nanomeshes [10], or substituting vacancy-site carbon atoms by nitrogen atoms [11]. From the perspective of the spintronics application of a 2-dimensional film, the former is superior to the latter, since it does not need to damage the graphene sheet and can maintain the integrity of the film. However, a theoretical prediction has not been experimentally verified yet: is it indeed universal to introduce magnetic moments in graphene by covalent sp^3^-type defects [12]?

In fact, atomic-scale control of the distribution of H atoms to paint magnetism on graphene has been achieved by scanning tunneling microscopy (STM) [13]. Graphene has also been proved to turn from diamagnetic to paramagnetic by fluorination [4]. It is undoubted that both H and F are effective at inducing magnetism in graphene and beyond that, only the effectiveness of hydroxyl-functionalized sp^3^-type defects has been reported [2]. However, the existing reports indicate that hydroxyl-functionalization of graphene can only be achieved by further processing of graphene oxide (GO), which needs a strong oxidant such as potassium permanganate in Hummers’ method [2] or potassium dichromate in Brodie’s method [14] because graphene is inert and hard to be chemically functionalized. That is to say, magnetic metal impurities such as manganese or chromium will have to be brought into hydroxyl-functionalization. To make sure the magnetic signals are intrinsic to graphene while not from the magnetic pollutants, repetitious washing of hydroxyl-functionalized graphene or GO with acid and deionized water must be done. Even so, it is still hard to guarantee all the 3-D contaminants are completely disposed of. Namely, hydroxyl sp^3^-defect may be not a good choice to experimentally confirm the universal validity for inducing magnetism in graphene. Amino group (NH_2_) provides a better choice than the hydroxyl group. 

In fact, there are a lot of ways to introduce highly atomic N in graphene [15,16]; however, the N types obtained are generally in-plane and there is no evidence they can generate amino sp^3^-type defects on the graphene basal plane. It has been reported that illuminating graphene under white light in ammonia atmosphere is a feasible way to form amino-type sp^3^-defects on the graphene basal plane [17,18]. By such a method, we can obtain a sufficient amount of sp^3^-type graphene suitable for measurement on a superconducting quantum interference device (SQUID) without importing original 3-D metals, as long as graphene is not originated from GO and can be massively produced—Parvez et al. provided a good way to solve such a problem by electrolytic exfoliation of graphite [19]. Graphene material is also prospective in high-precision low magnetic measurements using new switching sensing devices, which has high sensitivity, and compensate temperature drift [20,21].

In this study, we illuminated electrolyzed graphene (EG) with a decent few-layer ratio in ammonia to successfully obtain sp^3^-type N-doped graphene (sp^3^-NG). Our results demonstrate that almost all the N atoms are bonded to graphene basal-plane carbon atoms in the form of amino groups, and these amino-type sp^3^-defects can effectively introduce magnetic moments in graphene with an efficiency of ~1 μ_B_/100 NH_2_. We firstly experimentally proved the universal validity of inducing magnetic moments by amino-type sp^3^-defects and provide another potential candidate to prepare magnetic graphene, which is regarded as significantly crucial in the application of graphene spintronics. Furthermore, by using such sp^3^-type magnetic graphene to introduce localized magnetic moments on graphene, controlling the spin scattering to control the magnetoresistance as dilute F-doped sp^3^-functionalized graphene [22] is hopeful, and a potential alternative of light-element magnet [23] can be expected as well.

## 2. Experimental Section

### 2.1. Preparation

The graphene sheets were obtained through electrolytic exfoliation [19] of commercial graphite rods (99.999% purity, Beijing Gaochun, Beijing, China). Both electrodes were adopted as graphite rods and the electrolyte was ammonium sulfate solution with the concentration of 0.1 M. The distance between the two rods was 2 cm. The electrolytic voltage between the two poles was kept as 10 V and the initial output power was set as 10 W. Next, the exfoliated graphene sheets were collected with a PTFE membrane filter (0.2-μm pore size) by vacuum filtration and then washed by deionized water for 10 times. The graphene sheets were further exfoliated through ultrasonication in alcohol for 10 min at low power, and after 24 h of standing, only the supernatant was kept to render the sheets with a high few-layer ratio. To avoid any possible magnetic contaminants, the as-prepared graphene was washed by dilute nitric acid once and then by deionized water three times. Finally, after drying it in an oven at 60 °C, the original EG sample was successfully prepared.

Amino-functionalized sp^3^-NG was obtained by irradiating EG for 30 min with light from a 500 W high-pressure Hg lamp in NH_3_ (99%) atmosphere at a rate of 80 sccm [17]. For comparison, we also heat EG for 1 h at 200 °C under Ar atmosphere to get thermally treated graphene (TG) and under NH_3_ atmosphere to get nitrogen-doped graphene (NG), respectively.

### 2.2. Instrumentation

The morphologies of the samples were characterized using transmission electron microscopy (TEM, JEM-2100F, JEOL, Tokyo, Japan), and the X-ray photoelectron spectroscopy (XPS) measurements were performed on ESCALAB 250Xi (TMAG, Waltham, MA, USA) using an Al Kα radiation. Raman spectra were performed on Renishaw inVia (Wotton-under-Edge, UK) using a laser excitation of 532 nm. The magnetic properties of the samples were measured using SQUID magnetometer with a sensitivity less than 10^−8^ emu (Quantum Design MPMS-XL, San Diego, CA, USA), and all data was corrected for the diamagnetic contribution by subtracting the corresponding linear diamagnetic background at room temperature. The 3-D impurity elements of all the samples are measured by inductively coupled plasma (ICP) spectrometry (Jarrell-Ash, Waltham, MA, USA).

## 3. Results and Discussion

**Figure 1:**
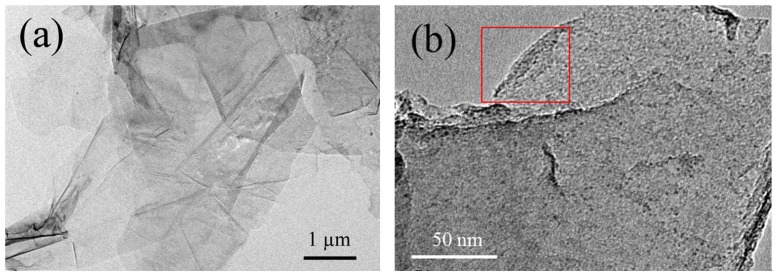
Typical TEM images of EG. The scale bar is (**a**) 1 μm and (**b**) 50 nm. In the red rectangular zone, the curved edge indicates the graphene sheet is few-layered.

Shown in Figure 1 are the typical TEM images of EG. It is easily found that the graphene sheets obtained by electrolytic exfoliation maintain two-dimensional ultrathin flexible structure and μm scale integrity, and are quite different from GO sheets with many ripples [2], the EG sheets are much flatter, maybe because there is no violent oxidation process during the preparation as GO has. From the curved edge of EG (see the red rectangle in Figure 1b), one can find a three- or four-layered graphene sheet, which means that by such kind of electrolytic exfoliation, few-layered graphene can be successfully obtained. Here we have to point out that, in the previous report [19], Parvez et al. got high-ratio mono-layered graphene through graphite electrolysis; however, it seems impossible to separate the mono-layered graphene sheets with sufficient quantity suitable for SQUID measurement, because we need at least several milligrams for each measurement to ensure the magnetic signals of the graphene samples are not completely flooded by the background signals.

**Figure 2:**
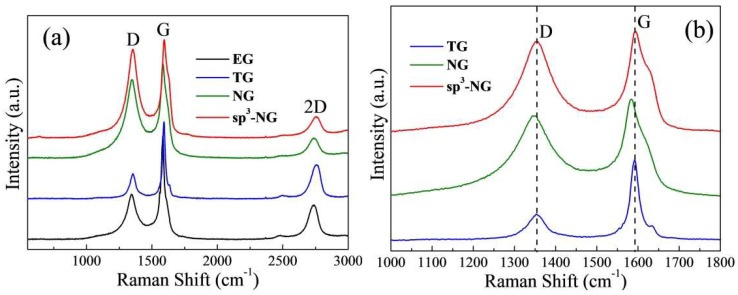
(**a**) Raman spectra of EG, TG, NG, and sp^3^-NG; (**b**) The D and G peaks of TG, NG, and sp^3^-NG.

The main features in the Raman spectra (Figure 2a) of carbon-based materials are the D, G and 2-D peaks that center at around 1350, 1580 and 2700 cm^−1^, respectively. The shape and height of 2-D peak is similar to the previous report [19], typically characteristic of electrolytic few-layered graphene sheets [24]. Obviously, EG sheet cannot maintain the pristine sp^2^-carbon-based structure of graphite or graphene since the D peak is prominent (*I_D_*/*I_G_* = 0.44), which demonstrates that during the electrolysis a lot of defects had been induced on the graphene sheet. By the determination of XPS measurement (Table 1), we found that EG has an oxygen atomic ratio of 12.1 at %, implying some oxygen groups are physically or chemically adsorbed on [19]. After thermally heating, TG has a distinctly lower D peak (*I_D_*/*I_G_* = 0.32) and oxygen content (5.5 at %), which means a lot of oxygen groups were removed and the pristine sp^2^-aromatic structure was restored to a certain extent. From Figure 2b, one can find that the G peak of TG is located at ~1585 cm^−1^, that is, with respect to 1580 cm^−1^ of pristine graphene, such blue shift means a lot of non-crystalline fractions still exist after heating [25]. It also can be seen in Figure 2b, by doping N through either thermal treatment (NG) or light treatment (sp^3^-NG), the defect ratios are dramatically increased (*I_D_*/*I_G_* is 0.83 for NG and 0.90 for sp^3^-NG, respectively). Note although the nitrogen contents of NG and sp^3^-NG are very close (Table 1), the D peak of sp^3^-NG is more prominent and the slight red-shift (~2 cm^−1^) of G peak of NG imply they may have different N-doping types. Since in-plane N atoms implanting into graphene will lead to the red shift of the G peak, we may guess the N-doping type of sp^3^-NG is out of plane, that is, amino sp^3^-type [17]. Moreover, the Raman spectrum of sp^3^-NG is very alike to lightly sp^3^-functionalized F-doping few-layered graphene [26]. The following XPS measurements confirmed our guess.

**Figure 3:**
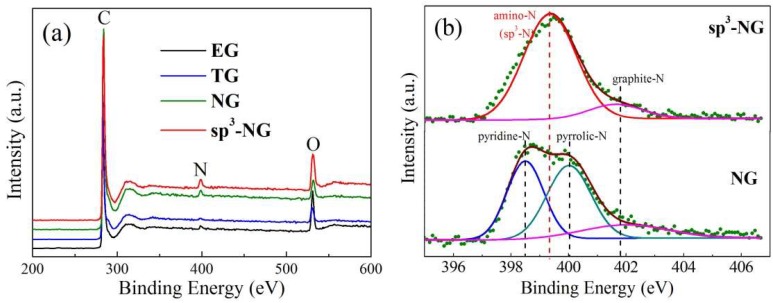
(**a**) XPS spectra of EG, TG, NG, and sp^3^-NG; (**b**) Typical fine-scanned XPS spectra of N 1 s of NG and sp^3^-NG. Blue, green, magenta, and red subpeaks are ascribed to pyridinic-, pyrrolic-, graphite- and amino-type (sp^3^-type) N–C bonding, respectively.

From the XPS spectra in Figure 3a, one can find that nitrogen peaks are almost invisible in EG and TG while evident in NG and sp^3^-NG, so we know that by thermal or light treatment EG in ammonia, the nitrogen atoms can be successfully inserted into the carbon skeleton of graphene. To identify the difference of the N-bonding environments of these two kinds of nitrogen doping, we carefully deconvolute the fine-scanned N 1s spectra of NG and sp^3^-NG (Figure 3b). Fairly interestingly, the sp^3^-NG sample shows nearly only a single strong peak which located at ~399.4 eV, typically manifested as amino-type N bonding to carbon (NH_2_–C) [17], accompanied with a very weak graphite-N subpeak located at ~401.7 eV. Unlike sp^3^-NG, aside from a similar weak graphite-N subpeak, NG presents another two subpeaks which sit at ~398.3 and 400 eV, typically identified as pyridinic- and pyrrolic-N, respectively. As is known, pyridinic-, pyrrolic- and graphite-N are all in-plane in graphene sheet while amino-N is out of plane to form sp^3^-defect, so we name the illuminated N-doped graphene as sp^3^-NG. As shown in Table 1, according to the deconvoluted subpeak area, we calculated the different N-type ratios and found that NG has a total N content of 2.6 at % with a proportion of pyridinic-N:pyrrolic-N:graphite-N ≈ 2:2:1, and sp^3^-NG is almost purely amino-N functionalized with a total N content of 2.9 at %. Naturally, the different types of N bonding will lead to different physical properties of N-doped graphene.

**Figure 4:**
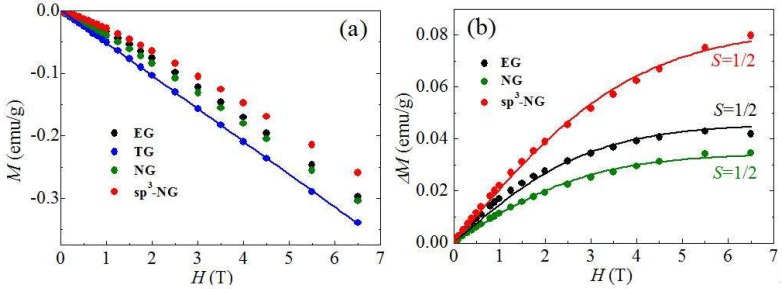
(**a**) Mass magnetization dependences on the applied magnetic field (*M*–*H*) of EG, TG, NG, and sp^3^-NG. The dots are the measurements and the blue solid line is linearly fit to the blue dots; (**b**) The dependences of mass magnetization of EG, NG and sp^3^-NG by subtracting which of TG on applied magnetic field (Δ*M–H*). The solid lines are fit to Brillouin function with *g* = 2. The measurement temperature is 2 K.

All the samples were compressed into a diamagnetic plastic bag for magnetism measurement. To make the results as accurate as possible, we used at least 20 mg samples for measurement of each run. Figure 4a shows the relationship of the mass magnetization (*M*) and the different applied magnetic field (*H*) of the typical samples under the temperature of 2 K. EG, TG, NG and sp^3^-NG are all diamagnetic, which means the intrinsic diamagnetism of graphene is predominant in these samples. However, the diamagnetic degrees are different. The diamagnetic measurements of TG (blue dots in Figure 4a) can be perfectly fitted with a line (blue line in Figure 4a), while EG, NG and sp^3^-NG have paramagnetic signals mixed in the diamagnetic signals. Based on the Experimental procedure and XPS measurements, it is easily known that TG is obtained by thermal treatment of EG and has lower oxygen content (see Table 1), indicating after the heating a lot of oxygen groups physiosorbed or chemisorbed on EG had been removed, so TG can be seen as a purer graphene sample than EG. Namely, the magnetic signals of TG can be taken as the pristine signals of diamagnetic graphene. Therefore, we can assess the magnetic moments induced by heteroatoms in EG, NG and sp^3^-NG by performing the subtraction of the magnetization of TG (Δ*M*). We plotted Δ*M* under different *H* in Figure 4b. Apparently, due to the positive Δ*M* of EG, NG and sp^3^-NG, both O and N heteroatoms have successfully introduced magnetic moments in graphene. To fit the Δ*M*–*H* curves with Brillouin function

(1) $$\Delta M=\Delta M_{s}[\frac{2S+1}{2S}Coth(\frac{2S+1}{2S}x)-\frac{1}{2S}Coth(\frac{x}{2S})]$$

where saturated magnetization $\Delta M_{s}=NgS\mu_{B}$, $x=gS\mu_{B}H/(k_{B}T)$, *k_B_* is the Boltzmann constant, *N* is the number of present magnetic moments, *S* is the spin angular momentum number, and *g* is the Landau factor assumed to be 2, we found that, all EG, NG and sp^3^-NG can be well fitted by using S=1/2, exhibiting the typical spin-1/2 paramagnetic behaviors of single point defects [4]. Correspondingly, Δ*M_S_* of EG, NG and sp^3^-NG are 0.045, 0.032 and 0.082 emu/g, respectively.

To analyze the magnetic sources of these samples, first, we must make certain the magnetic moments are not originated from 3-D contaminants. Through ICP measurement, the possible impurity contents are listed in Table 2, and one can find that their contents are trivial enough to be ignored. For instance, the highest Fe content in EG is 0.61 ppm, and it can generate the maximal magnetization of ~4 × 10^−4^ emu/g, which is far less than the magnetization we got (10^−2^ order of magnitude). Note that we burned at least 30 mg sample each time for ICP measurement, which avoided the influence from the uneven distribution of 3-D metals. Thus, we can further study the magnetic sources without considering possible extrinsic factors. Previous reports [2,5,27,28] indicate that adsorbed heteroatoms on the graphene basal plane can produce magnetic moments, for the *p_z_* orbitals of carbon atoms are partly occupied to change the symmetric structure of π electron system. That is, uncompensated electron spins are generated. During the electrolysis, due to the decomposition of water, many oxygen atoms are physically adsorbed on or even form hydroxyl groups to bond to the graphene basal plane [19], thus inducing magnetic moments in EG. After heat treatment, some oxygen atoms were released, or unstable oxygen groups were decomposed, the bipartite honeycomb lattice of graphene was partly recovered and hence, some magnetic structures were converted back into non-magnetic states. As a result, TG is more diamagnetic than EG. What we are more interested is the magnetic moments induced by N atoms. However, the magnetization of NG is even lower than EG, implying the N atoms in NG did not introduce remarkable magnetic moments in graphene. Different from NG, sp^3^-NG has an improvement of magnetization to about twofold of EG, indicating the N atoms in sp^3^-NG are effective to induce magnetic moments. Clearly, the different types of N atoms resided on NG and sp^3^-NG bring forth such different effects.

**Figure 5:**
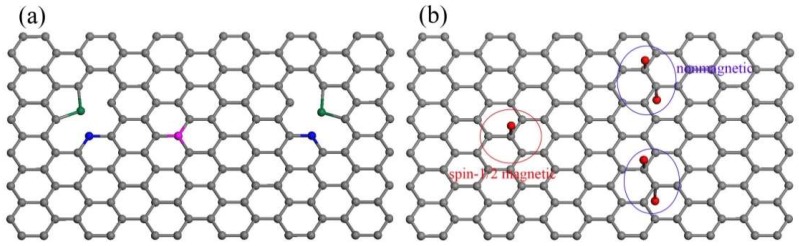
Schematic representation of (**a**) NG and (**b**) sp^3^-NG. Carbon atoms are grey. Pyridinic-, pyrrolic- and graphite-N atoms are blue, green, and magenta, respectively. The red balls denote the amino groups (-NH_2_) covalently bonded to the basal-plane carbon atoms. In the red oval region there is an isolated amino group and in blue ones two amino groups chemisorbed on different sublattices to form AB dimers.

According to the XPS deconvolution results (Figure 3b), possible N types of NG and sp^3^-NG were schematically represented in Figure 5. It is known that all the in-plane pyridinic-, pyrrolic- and graphite-N atoms can produce magnetic moments in carbon materials due to the extra electrons of N both experimentally and theoretically [11,28,29,30], but they face two big problems: (i) pyridinic- and pyrrolic-N can only exist at the vacancy- or edge-site of graphene to form highly active dangling bonds, therefore they are easily bonded to other atoms and the extra electrons are consequently covalently paired to lose the magnetic moments [31]; and (ii) the contents of graphite-N are generally extremely low in N-doped graphene. Up till now it has been hard to experimentally testify its effectiveness of inducing magnetic moments in graphene. For this reason, our NG sample is weakly paramagnetic (after subtraction of pristine diamagnetic signals of TG, see Figure 4b), and it is even weaker than EG—in other words, the magnetic moments induced by pyridinic-, pyrrolic- and graphite-N is even less than adsorbed oxygen groups. We also implemented thermally doping N at a higher temperature (500 °C)—generally it’s seen as the most effective temperature to dope more N and results in higher magnetization—and found that the magnetization of NG is even lower than that which was thermally N-doped at 200 °C. To sum up, in-plane N atoms did not bring in any noteworthy effect of inducing magnetic moments in our graphene samples.

As for an isolated amino-N, it creates a sp^3^-type point defect on graphene basal-plane to introduce the magnetic moment of 1 μ_B_ (see red oval region in Figure 5b), without considering forming dangling bonds. Only when the nearest neighbor site is simultaneously occupied by another amino group (see blue oval region in Figure 5b) or when two adjacent sp^3^-defects sit on the different sublattices (AB dimer) [12,13], will it be non-magnetic. Moreover, several near sp^3^-defects sitting on the same sublattice can contribute large spin clusters (e.g., AA dimer and AAA trimer contribute 2 and 3 μ_B_, respectively) [13]. However, the coupled spin clusters were not found in our sp^3^-NG samples since its Δ*M* exhibit good spin-1/2 paramagnetic behavior (Figure 4b), so we can speculate the amino groups on sp^3^-NG are all isolated or AB dual. The saturated Δ*M* is 0.082 emu/g, which means the efficiency of inducing magnetic moments in graphene by amino-type sp^3^ defects is ~1 μ_B_/3000 C or 1 μ_B_/100 NH_2_. Such an efficiency is close to fluorine-doped sp^3^-defects (2–20 μ_B_/1000 F) [4]. We tried to alter the amino coverage on the graphene basal-plane to tune the magnetism by changing the illumination time, but it cannot work because in a very short time (~1 min) the photochemical N-doping will nearly saturate and lose the ability of enhancing amino coverage. Anyway, as a sp^3^-type defect, the amino group exhibits the universal effectiveness of inducing magnetic moments in graphene.

## 4. Conclusions

In summary, we have prepared almost purely amino-functionalized graphene by white-light illumination of EG. As a sp^3^-type defect, the amino group can introduce magnetic moments in graphene with an efficiency of 1 μ_B_/100 NH_2_. As is known, the existence of localized magnetic moments is a prerequisite for magnetic coupling to induce ferromagnetism in graphene, which is deemed promising to design a spin field-effect transistor (SFET). Although the as-prepared amino-functionalized graphene is still diamagnetic, the validity of inducing magnetic moments of amino groups provides the imagination space to achieve ferromagnetic graphene. Moreover, unlike F atoms producing lots of holes on graphene basal plane to damage the integrity of graphene film when generating sp^3^-defects [32], the photochemical process to dope amino groups on graphene is facile and keeps the completeness of 2-dimensional film, and thus, magnetic amino-functionalized graphene is more advantageous in SFET when film integrity is required. In short, the universal validity of spin generalization in graphene by sp^3^-type defect was verified experimentally, laying a solid foundation for graphene magnetism theory, and paving the way for its potential applications in spintronics.

## Figures and Tables

**Table 1:** The ratios of different types of N and elemental contents of the typical samples.

Samples (at %)	Pyridinic-N	Pyrrolic-N	Graphite-N	Amino-N	N	O	C
EG	-	-	-	-	0.8	12.1	87.1
TG	-	-	-	-	0.7	5.5	93.8
NG	1.0	1.1	0.5	0	2.6	4.6	92.8
sp^3^-NG	0	0	0.3	2.6	2.9	8.5	88.6

**Table 2:** The contents of the typical 3-D metal impurities of the samples. The unit is ‘ppm’. ‘ND’ denotes ‘not found’.

Impurities	Fe	Co	Ni	Cr	Mn	Al
EG	0.61	ND	0.07	0.05	0.08	0.25
TG	0.32	ND	0.03	0.04	0.09	0.16
NG	0.31	ND	0.06	0.05	0.10	0.20
sp^3^-NG	0.50	ND	0.03	0.04	0.09	0.12
